# [Expression of Concern] Podoplanin-mediated TGF-β-induced epithelial-mesenchymal transition and its correlation with bHLH transcription factor DEC in TE-11 cells

**DOI:** 10.3892/ijo.2025.5805

**Published:** 2025-09-25

**Authors:** Yunyan Wu, Qiang Liu, Xu Yan, Yukio Kato, Makiko Tanaka, Sadaki Inokuchi, Tadashi Yoshizawa, Satoko Morohashi, Hiroshi Kijima

Int J Oncol 48: 2310-2320, 2016; DOI: 10.3892/ijo.2016.3445

Following the publication of the above paper, it was drawn to the Editor's attention by a concerned reader that, for the scratch-wound assay experiments shown in Fig. 6 on p. 2318, the 'control siRNA/24 h' and 'podoplanin siRNA/48 h' panels contained an overlapping section of data; such that these data, which were intended to show the results from differently performed experiments, appeared to have been derived from the same original source. Upon analyzing the data independently in the Editorial Office, it came to light that, in addition to control blots, the podoplanin blots were duplicated in Fig. 2A and B, and also in Fig. 3A and B, although it wasn't clear whether this was simply the way in which the authors had chosen to arrange the data in these figures, as the reported experimental conditions were the same in the respective figure parts.

The authors were contacted by the Editorial Office to offer an explanation for these possible anomalies in the presentation of the data in this paper, although up to this time, no response from them has been forthcoming. Owing to the fact that the Editorial Office has been made aware of potential issues surrounding the scientific integrity of this paper, we are issuing an Expression of Concern to notify readers of this potential problem while the Editorial Office conitnues to investigate this matter further.

